# Green Extraction Methods for Extraction of Polyphenolic Compounds from Blueberry Pomace

**DOI:** 10.3390/foods9111521

**Published:** 2020-10-23

**Authors:** Ante Lončarić, Maria Celeiro, Antun Jozinović, Josip Jelinić, Tihomir Kovač, Stela Jokić, Jurislav Babić, Tihomir Moslavac, Sandra Zavadlav, Marta Lores

**Affiliations:** 1 Faculty of Food Technology Osijek, Josip Juraj Strossmayer University of Osijek, Franje Kuhača 18, HR 31000 Osijek, Croatia; antun.jozinovic@ptfos.hr (A.J.); jjelinic@ptfos.hr (J.J.); tihomir.kovac@ptfos.hr (T.K.); stela.jokic@ptfos.hr (S.J.); jurislav.babic@ptfos.hr (J.B.); tihomir.moslavac@ptfos.hr (T.M.); 2 CRETUS Institute, Department of Analytical Chemistry, Nutrition and Food Science, Universidade de Santiago de Compostela, E-15782 Santiago de Compostela, Spain; maria.celeiro.montero@usc.es (M.C.); marta.lores@usc.es (M.L.); 3 Department of Food Technology, Karlovac University of Applied Sciences, Trg J. J. Strossmayera 9, 47000 Karlovac, Croatia; sandra.zavadlav@vuka.hr

**Keywords:** high voltage electrical discharges, pulsed electric field, ultrasound, blueberry pomace, polyphenolic compounds

## Abstract

In this study, green extraction methods—high voltage electrical discharges (HVED), pulsed electric field (PEF), and ultrasound-assisted extraction (UAE)—were compared in terms of extraction yield of total and individual polyphenolic compounds, as well as the antioxidant capacity of blueberry pomace extracts. All extractions were performed with methanol- and ethanol-based solvents. The highest total polyphenols content (TPC) (10.52 mg of gallic acid equivalent (GAE) per g of dry weight (dw)) and antioxidant activity (AA) (0.83 mmol TE/g dw) were obtained by PEF-assisted extraction in the ethanol-based solvent after 100 pulses and 20 kV/cm, which corresponds to an energy input of 41.03 kJ/kg. A total of eighteen individual polyphenols were identified in all investigated blueberry pomace extracts by high-performance liquid chromatography with the diode-array detector (HPLC-DAD) and liquid chromatography electrospray ionization tandem mass spectrometric (LC-(HESI)-MS/MS). The highest anthocyanin (1757.32 µg/g of dw) and flavanol (297.86 µg/g of dw) yields were obtained in the methanol-based solvent, while the highest phenolic acid (625.47 µg/g of dw) and flavonol (157.54 µg/g of dw) yields were obtained in the ethanol-based solvent by PEF-assisted extraction at the energy input of 41.03 kJ/kg. These results indicated that PEF is a promising green extraction method which can improve the blueberry pomace’s polyphenol extraction yield.

## 1. Introduction

Blueberries are very popular fruit due to their numerous health benefits, nutritional value, and excellent sensory evaluation. They belong to the group of highly perishable soft fruits with a short shelf life [1]. To prolong the consumption of blueberries throughout the year, they can be preserved or processed into different products. More than 50% of fresh blueberries are processed into some kind of final fruit product; the most important commercial product is juice [2]. During juice production, waste generation may be up to 20% of the initial fruit weight [3]. Blueberries contain high amounts of anthocyanins, phenolic acids, flavanols, and flavonols [4], and the evident fact is that these compounds have shown pharmacological benefits for the prevention of various chronic diseases such as cancer, diabetes, hypertension, and hypercholesterolemia [5,6,7,8]. It was reported that blueberry pomace represents a rich source of named phenolic compounds with an important antioxidant capacity [9]. Nowadays, consumers’ awareness of functional foods and the importance of polyphenols and antioxidants in the diet increased demand for food quality. To respond to these demands, the food industry is facing the challenge of ensuring more natural and safe sources of functional ingredients. Fruit and vegetable by-products are the main sources of natural polyphenolic compounds, and extraction is considered the best approach for the valorisation of these compounds [4].

Common extraction methods such as maceration, digestion, Soxhlet extraction, and others usually involve large amounts of solvent, are time consuming, and can cause degradation of some of the desired compounds. That is why such methods are considered non-green [10]. For that reason, there is a trend in exploring novel and green methods for the extraction of polyphenol compounds. The goal for the exploring of novel extraction techniques is to shorten the extraction time, reduce energy consumption and negative environmental impact, increase safety as well as enhance innovation and competitiveness [11]. In this way, high voltage electrical discharges, pulsed electric field, or ultrasound energy have recently been proposed to shorten the processing time, increase recovery yield, and enhance the functionality of extracts [12]. 

High voltage electrical discharges (HVED) are one of the innovative liquid phase discharge technologies. The principle of HVED action is based on two phases: the corona streamer discharge process known as the pre-breakdown phase and the arc discharge process known as the breakdown phase. During the pre-breakdown phase, the relatively weak shock waves and formation of a small number of little bubbles can be observed. Besides that, strong UV radiations and active radicals are also generated; however, if electric field intensity is moderate, it will influence only cell destruction efficacy rather than oxidase compounds such as polyphenols [13]. The enhanced electrohydraulic phase occurs during the transition of pre-breakdown to breakdown phase, causing several effects: strong shock waves, strong UV radiations, production of highly concentrated free radicals, bubbles with plasma inside, and strong liquid turbulence [13,14,15]. The named effect can cause product fragmentation, mechanical destruction of cell tissues as well as cell oxidation [16]. The HVED has been applied for the extraction of polyphenols from wine by-products [17,18,19], pomegranate peel [20], peanut shells [21], papaya peel [22], etc.

Pulsed electric field technology (PEF) is based on electroporation phenomena. Strictly speaking, putting the cell in a strong electric field causes accumulation of oppositely charged ions on both sides of the membrane, when transmembrane potential exceeds a critical value of approximately 1 V, the repulsion between charge-carrying molecules leads to membrane thickness reduction and permeabilization to small molecules [15]. Depending on electric field strength and treatment intensity, the permeabilization could be reversible or irreversible depending on the purpose of treatment [23]. To achieve good extraction results in soft plant tissues or materials, such as the mesocarp or pericarp of most fruits, electric field strengths between 0.1 and 10 kV/cm should be employed. However, electric field strength up to 20 kV/cm should be employed in order to achieve good extraction results from e.g., seeds and stalks where lignification can occur [24]. PEF has been used for the extraction of polyphenols from blueberry processing by-products [14], orange peel [25], purple-fleshed potato [26], grape seed [27], and grape by-products [28].

Ultrasound-Assisted Extraction (UAE) is a widely accepted extraction technique for polyphenol recovery from fruit by-products [29]. The UAE stands out as a sustainable alternative due to simplicity, inexpensiveness, and higher efficiency compared to conventional methods such as maceration. UAE is based on the principle of acoustic cavitation, which involves the formation, growth, and implosion of bubbles damaging the cell walls of the plant matrix and thereby favouring the release of bioactive compounds [30]. This technology has been studied quite extensively for the recovering of polyphenols from different fruit wastes, among which are blueberry by-products [4,31,32].

For the extraction of polyphenols, the most used organic solvents are ethanol and methanol. In food systems, ethanol, considered as environmentally friendly and generally recognised as a safe (GRAS) solvent for the European Food Safety Authority (EFSA), is preferred because of several advantages: it is completely biodegradable, available in high purity form, low cost, and abundance, in addition to being compatible with health [16] to some extent.

This study aimed to compare the differences in yield of total and individual polyphenolic compounds and antioxidant activity of blueberry pomace extracts obtained by different methods: high voltage electrical discharges (HVED), pulsed electric field (PEF), and ultrasound-assisted extraction (UAE).

## 2. Materials and Methods

### 2.1. Chemicals

Methanol (employed as an extraction solvent and as mobile phase), and ultrapure water (for mobile phase preparation), both LC-MS grade, were supplied by Scharlau (Chemie S.A., Barcelona, Spain). Deionised water was produced in the laboratory with a MilliQ gradient system (Millipore, Bedford, MA, USA), while formic acid was supplied by Merck (Darmstadt, Germany). All other chemicals, such as ethanol, Folin–Ciocalteu reagent or hydrochloric acid (HCl), were purchased from Sigma-Aldrich (Chemie GmbH, Steinheim, Germany). Catechin (CAS: 154-23-4, ≥99.0%) procyanidin B1 (CAS: 20315-25-7, ≥90%), caffeic acid (CAS 331-39-5, ≥98.0%), procyanidin B2 (CAS: -49-8, ≥90%), chlorogenic acid (CAS: 327-97-9, ≥95%), epicatechin (CAS: 490-46-0; ≥98%), 4-hydroxycinnamic acid (CAS: 7400-08-0, ≥98.0%), myricetin (CAS: 529-44-2, ≥96.0%), quercetin (CAS: 117-39-5, ≥95%), kaempferol (CAS: 520-18-3, ≥97.0%), delphinidin (CAS: 528-53-0, ≥95%), petunidin (CAS: 1429-30-7, ≥90%), cyanidin (CAS: 528-58-5, ≥98%), peonidin (CAS: 134-01-0, ≥95%), and malvidin (CAS: 643-84-5, ≥95%) were purchased from Sigma-Aldrich (Merck KGaA, Darmstadt, Germany).

### 2.2. Plant Material

Blueberry pomace was obtained after juice production. The fresh blueberries were a gift from a plantation in Mikleuš (Bogadi d.o.o., Mikleuš, Croatia). Lyophilisation of blueberry pomace was carried out in an Alpha LSCplus (Christ, Osterode am Harz, Germany) freeze-dryer at a temperature range from −80 to 25 °C under 0.180 mbar. The final drying or isothermal desorption was carried out at 25 °C and a pressure of 0.060 mbar. After freeze-drying, blueberry pomace was pulverised in a laboratory mill (Universal mill M20, Buch & Holm, city, Herlev, Denmark) to a stabilised form and preserved in amber bottles at 4 °C until analysis (7–10 days). The total solid content of freeze-dried blueberry pomace was 98%.

### 2.3. Polyphenols Extraction

For the extraction of polyphenols from blueberry pomace, extractions were performed using two different hydro-organic solvent systems (50% of ethanol and 1% HCl or 50% of methanol and 1% HCl). The extractions were carried out with a liquid to solid ratio of 50:1 mL/g.

### 2.4. High Voltage Electrical Discharge-Assisted Extraction (HVED)

**Figure 1:**
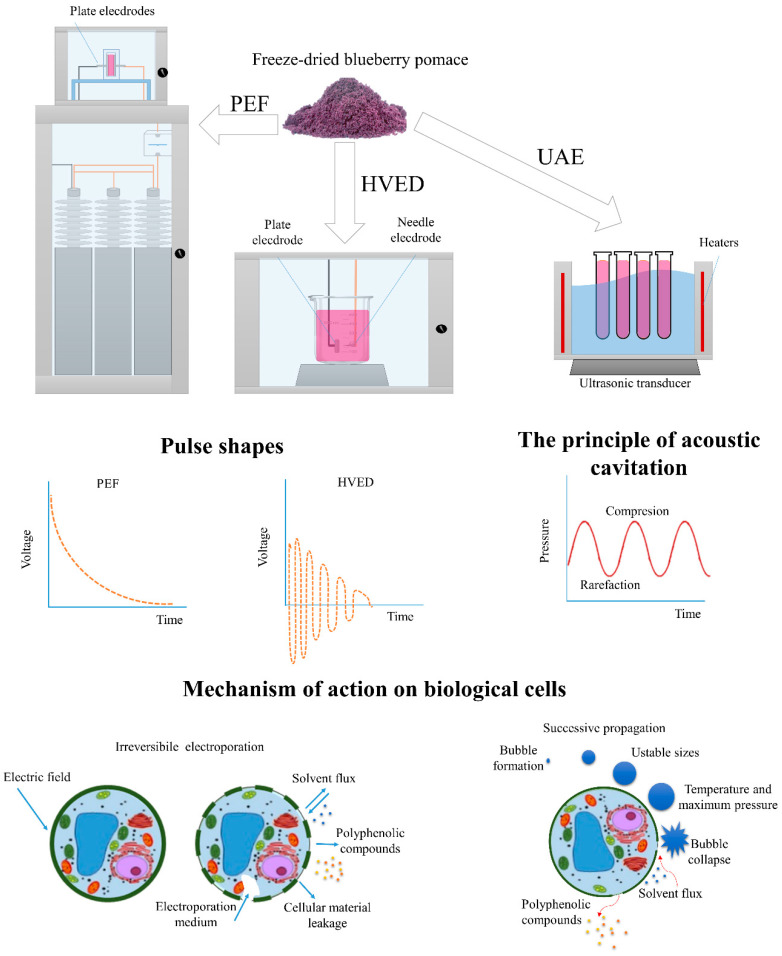
Example of processing chambers, pulse shapes used, and mechanism of action of used extraction techniques. PEF—pulsed electric field; HVED—high-voltage electrical discharge; UAE—ultrasound-assisted extraction.

The HVED experiments were performed in a laboratory treatment chamber connected to a pulsed high-voltage power supply (Inganiare CPTS1, Osijek, Croatia), shown in Figure 1. The electrical discharges were generated by an electrical breakdown in solvent systems. The high-voltage pulse generator provided the 30 kV discharges in a 600 mL chamber with electrodes of a needle-plate geometry. A sharpened stainless steel needle with a diameter of 2.5 mm was used. A positive pulse voltage was applied to the stainless steel cylindrical needle (diameter 2.5 mm) and as a ground electrode, a stainless disc (diameter 45 mm) was used with a distance between the electrodes of 5 mm. The treatment was conducted at room temperature (25 °C) and on a magnetic stirrer at different HVED frequencies (20, 50, and 100 Hz) and time (5, 10, and 15 min). The waveform, current, and voltage were indicated by an oscilloscope.

### 2.5. Pulsed Electric Field-Assisted Extraction (PEF)

The PEF experiments were performed in a laboratory treatment chamber containing parallel stainless steel electrodes (5 × 2 cm), shown in Figure 1. The electrode area was 10 cm^2^ and the distance between the electrodes was 1 cm. The treatment chamber was connected to a high-voltage pulse generator, providing up to 25 kV discharges in a 10 mL chamber. The energy was stored in a set of low inductance capacitors, which were charged by the power supply; a spark gape was used as a switch. The pomace was treated by an exponentially decaying pulse with a pulse duration of 2 μs. The oscilloscope indicated the voltage, waveform, and current. The specific energy input for both HVED (W_HVED_) and PEF (W_PEF_) was obtained from Equation (1):

(1) $$W=\frac{E_{P}\times n}{m}$$

where *W* is the energy input (kJ/kg), *Ep* is the energy of one pulse (kJ), and *m* is the mass of suspension (kg). The energy of one pulse (*Ep*_HVED_ and *Ep*_PEF_) was obtained from Equation (2):

(2) $$Ep=\int_{0}^{t}U\times I\times dt$$

where *U* is the voltage (V) and *I* is the current (A).

Electric field intensity (*E*_HVED_ and *E*_PEF_) was obtained from Equation (3)

(3) $$E=\frac{V}{d}$$

where *E* is the electric field intensity (kV/cm); *V* is the peak voltage (kV); *d* is the distance between electrodes (cm).

### 2.6. Ultrasound-Assisted Extraction (UAE) 

Ultrasound-assisted extraction (UAE) was performed in an ultrasonic bath (Figure 1) at 35 kHz at different temperatures (20, 40, and 80 °C) and times (5, 10, and 15 min) in 20 mL test tubes. After the correspondent extraction time, the obtained extracts were centrifuged (Heareus, Multifuge 3 L-R Centrifuge, Thermo Fisher Scientific, San Jose, CA, US) at 25 °C for 15 min and filtered through a 0.25 µm polytetrafluoroethylene (PTFE) syringe-tip filter (Chromafil Xtra, Macherey-Nagel GmbH & Co. KG, Düren, Germany).

### 2.7. Determination of Total Polyphenolic Content and Antiradical Activity

The total polyphenolic content (TPC) was determined by employing Folin–Ciocalteu reagent (FC) according to a procedure described by Singleton and Rossi [33]. Briefly, 0.2 mL of extract was mixed with 1.8 mL of deionised water, 10 mL of FC (1:10), and 8 mL of 7.5% of sodium carbonate in a test tube. The development of blue colour was monitored at 765 nm after 120 min. The TPC was quantified from the gallic acid calibration curve (1–20 mg/L, *R*^2^ = 0.9929). The TPC was calculated and expressed as mg gallic acid equivalent (GAE) per g of dry weight of blueberry pomace.

The antiradical activity (AA) was measured using a DPPH radical according to the methodology described by Brand-Williams [34]. The reaction mixture consisted of 0.2 mL of the extract and 3 mL of DPPH radical solution with 0.5 mM in ethanol. The changes in the colour of the radical from deep violet to light yellow were measured at 517 nm using a UV–vis spectrophotometer (Jenway 6300, Bibby Scientific, UK). The antiradical activity (AA) was quantified from the Trolox^®^ calibration curve (0.1–1.5 mmol TE/g, *y* = 0.9548*x* + 0.0294; *R*^2^ = 0.9937). The AA was calculated and expressed as millimoles of Trolox^®^ equivalents (TE) per gram of dry blueberry pomace (mmol TE/g dw).

### 2.8. High-Performance Liquid Chromatography with the Diode-Array Detector (HPLC-DAD)

**Figure 2:**
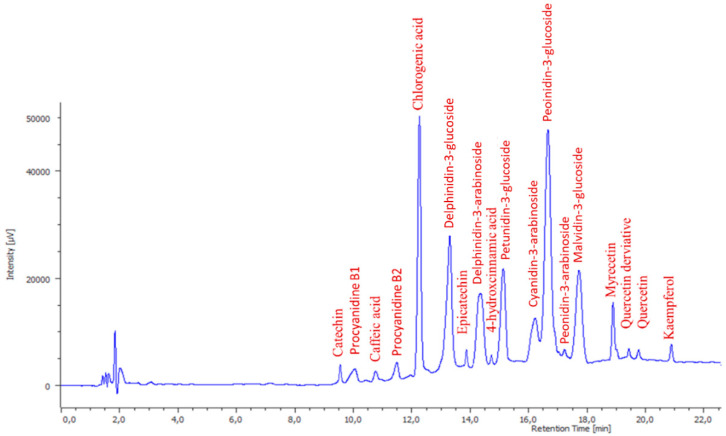
High-Performance Liquid Chromatography with the Diode-Array Detector (HPLC-DAD) chromatogram (280 nm) obtained for a blueberry pomace sample.

Polyphenol identification was performed on an HPLC Jasco LC Net II equipped with an AS-4150 autosampler, PU-4180 pump, and MD-4010 PDA detector. JASCO ChromNAV Version 2.01.00 (JASCO International Co., Ltd., Tokyo, Japan) was used for controlling the analysis. Separation of individual polyphenols was performed at 50 °C on a C18 Kinetex column (150 × 4.5 mm, 2.6 μm) (Phenomenex, Torrance, CA, USA). The mobile phase consisted of: solvent A—water and 1% of formic acid; B—methanol and 1% of formic acid. The gradient elution system was: 10 min, linear gradient from 95% A to 80%; 5 min, linear gradient from 80 to 70%; 5 min, linear gradient from 70 to 50%; 5 min, linear gradient from 50 to 0%; 10 min isocratic. The injection volume was 5 µL and the flow rate was 1 mL/min. The chromatograms were monitored in the range of 190 to 600 nm. Polyphenols were detected at 280 nm (catechin, *y* = 1496.9*x* − 31,807; *R*^2^ = 0.9995; epicatechin, *y* = 6591*x* − 2097.6; *R*^2^ = 0.9999; procyanidin B1, *y* = 3184.4*x* + 140,717; *R*^2^ = 0.996 and B2, *y* = 4661.8*x* − 1572.8; *R*^2^ = 0.9986), 320 nm (caffeic acid, *y* = 39,450*x* + 2 × 10^7^ *R*^2^ = 0.9953; chlorogenic acid, *y* = 16,186*x* − 891,959; *R*^2^ = 0.9956 and 4-hydroxycinnamic acid, *y* = 4004.7*x* + 724,691; *R*^2^ = 0.9972), 360 nm (myricetin, *y* = 4705.9*x* – 7 × 10^6^; *R*^2^ = 0.9944; quercetin, *y* = 2937.1*x* − 911,531; *R*^2^ = 0.9976 and kaempferol, *y* = 1279.5*x* + 189,066; *R*^2^ = 0.9974), and 520 nm (anthocyanins) (Figure 2) and identified by comparison of their retention times and UV–vis spectra to those of pure standards. Anthocyanins were tentatively identified by the help of the literature data [4,35]. Tentatively identified anthocyanins were quantified by using their aglycone calibration curves, delphinidin (*y* = 20,543*x* + 27,568; *R*^2^ = 0.9951), petunidin (*y* = 8486*x* + 13,533; *R*^2^ = 0.9984), cyanidin (*y* = 4265.8*x* + 280,378; *R*^2^ = 0.9959), peonidin (*y* = 271*x* + 28,509; *R*^2^ = 0.9914), and malvidin (*y* = 35,321*x* + 24,302; *R*^2^ = 0.9976). Quantification has been performed by external standard calibration and the calibration range for each phenolic standard was 0.1–10 µg/g. The amount of polyphenols was expressed as µg/g of dry weight (dw).

### 2.9. Phenolic Identification by LC-MS-MS

Identification of the major polyphonic compounds was performed by LC-MS/MS, employing a Thermo Scientific (San Jose, CA, USA) instrument based on TSQ Quantum Ultra^TM^ triple quadrupole mass spectrometer equipped with a heated electrospray ionisation (HESI-II) source and an Accela Open autosampler with 20 µL loop. The chromatographic separation was achieved on a Kinetex C18 column (2.6 μm, 100 × 2.1 mm) (Phenomenex) at 50 °C. The mobile phase was composed of A—water with 0.1% of formic acid; B—methanol, with 0.1% of formic acid. The gradient program started with 5% B (held 5 min); it was increased to 90% of B in 11 min and kept constant for 3 min. Finally, the initial conditions were reached in 9 min. The injection volume was 10 µL, and the mobile phase flow rated was 0.2 mL/min. Selected Reaction Monitoring (SRM) acquisition mode was employed, monitoring 2 or 3 MS/MS transitions per compound (Table 1). Polyphenols were detected in the negative mode (ESI-NI) and thus, producing mainly the [M − H]^−^ molecular ions (Table 1) except myricetin, quercetin, and anthocyanins which were detected in positive mode. For optimisation of the MS/MS transitions, the standard of the target polyphenol was introduced into the mass spectrometer by direct flow injection, and the collision energies of the SRM transitions were optimised for each polyphenol. The system was operated by Xcalibur 2.2. (Thermo Fisher Scientific Inc., San Jose, CA, USA) and Trace Finder 3.2. (Thermo Fisher Scientific Inc., San Jose, CA, USA) software.

### 2.10. Statistical Analysis

All extractions were performed in triplicate. Significant differences between the results were evaluated by analysis of variance (ANOVA) using Statgraphics XV Centurion software package (Manugistics Inc., Rockville, MD, USA). Differences at *p*-value < 0.05 were considered statistically significant at a 95% the confident level. The Least Significant Difference (LSD) test was applied to indicate the samples between which there were differences. For drawing of the Sankey diagram, Flourish studio was used (Flourish Studio, Kiln Enterprises Ltd., London, UK).

## 3. Results

### 3.1. Total Polyphenolic Content and Antioxidant Activity of Extracts

**Figure 3:**
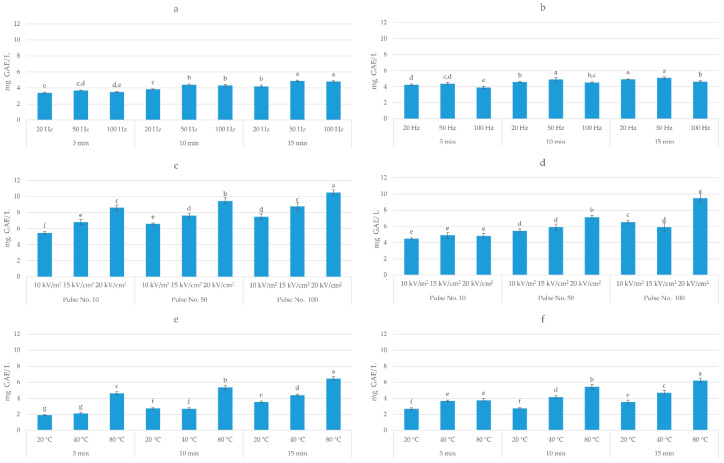
Total polyphenol content (mg GAE/g dw) of: (**a**) high voltage electrical discharges (HVED) ethanol-based extracts; (**b**) HVED methanol-based extracts; (**c**) pulsed electric field (PEF) ethanol-based extracts; (**d**) PEF methanol-based extracts; (**e**) ultrasound-assisted extraction (UAE) ethanol-based extracts; (**f**) UAE methanol-based extracts. Bars with different letters are significantly different (*p* < 0.05).

**Figure 4:**
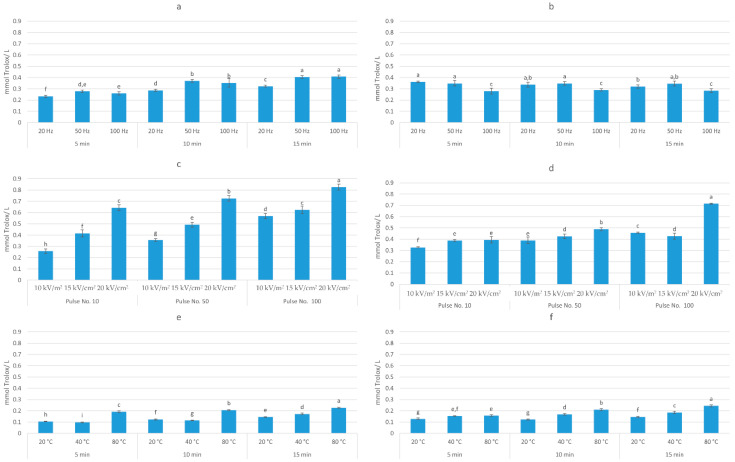
Antioxidant activity (mmol TE/g dw) of: (**a**) HVED ethanol-based extracts; (**b**) HVED methanol-based extracts; (**c**) PEF ethanol-based extracts; (**d**) PEF methanol-based extracts; (**e**) UAE ethanol-based extracts; (**f**) UAE methanol-based extracts. Bars with different letters are significantly different (*p* < 0.05).

The first objective of this study was to evaluate the impact of HVED, PEF, and UAE on the total polyphenol content and the antiradical activity of blueberry pomace extracts. The obtained results are presented in Figure 3 and Figure 4.

Three-way ANOVA analysis (solvent, time, and frequency) of HVED-assisted extraction showed that all investigated factors had a significant effect (*p* < 0.05) on the extraction of TPC. The highest yield (6.58 mg GAE/g dw) was obtained in methanol-based solvent after 15 min of treatment time and at the frequency of 50 Hz, which corresponds to an energy input of 22.27 kJ/kg. On the other hand, different trends were observed for AA, where only time and frequency had a significant effect (*p* < 0.05) on the antioxidant activity of obtained extracts. The highest AA (0.41 mmol TE/g dw) was achieved in ethanol-based solvent after 15 min of treatment time and at the frequency of 100 Hz. Three-way ANOVA analysis of PEF-assisted extraction showed that all investigated factors (solvent, electric field intensity, and number of pulses) had a significant effect (*p* < 0.05) on the extraction of TPC and AA of extract. The highest TPC (10.52 mg GAE/g dw) and AA (0.83 mmol TE/g dw) was obtained in ethanol-based solvent after 100 pulses and 20 kV/cm, which corresponds to the energy input of 41.03 kJ/kg. Considering UAE, the three-way ANOVA analysis showed that also all investigated factors (solvent, temperature, and time) had a significant effect (*p* < 0.05) on the TPC and AA of extract. The highest TPC (5.46 mg GAE/g dw) was obtained in ethanol-based solvent after 15 min at 80 °C and AA (0.25 mmol TE/ g dw) in methanol-based solvent after 15 min at 80 °C. Rajha et al. [36] also demonstrated the higher efficiency of electrical treatments over ultrasound, however, giving the advantage to HVED over PEF. In their study, for the same energy input, HVED enhanced the recovery of polyphenols by ≈3 and ≈1.3 times as compared with UAE and PEF, respectively. This may explain the higher yield of polyphenols obtained by PEF in this study, since the energy input of PEF was almost twice higher comparing to the energy input of HVED.

Table 2, Table 3, Table 4 and Table 5 present the results of individual polyphenols content determined in the blueberry extracts by HPLC-DAD and confirmed by LC-MS/MS.

### 3.2. Extraction of Anthocyanins

Anthocyanins were the most abundant polyphenolic group of compounds (Table 2) detected in the blueberry extract. Seven anthocyanins were identified in all extracts obtained in this study, regardless of the method and conditions investigated. They are delphinidin 3-glucoside, delphinidin-3-arabinoside, petunidin 3-glucoside, cyanidin-3-arabinoside, peonidin 3-glucoside, peonidin 3-arabinoside, and malvidin 3-glucoside. These anthocyanins have been previously detected in blueberries [4,37,38]. Peonidin 3-glucoside was the most abundant anthocyanin in the blueberry extract obtained in this study. The total anthocyanin content ranged from 285.73 to 1757.32 µg/g of dw, depending on the extraction solvent, extraction method, and method parameters. Three-way ANOVA analysis showed that all investigated factors (HVED—solvent, time, and frequency; PEF—solvent, electric field intensity, and number of pulses; UAE—solvent, temperature, and time) had a significant effect (*p* < 0.05) on the extraction of anthocyanins. Regarding the extraction solvent, the methanol-based solvent proved as a better extraction solvent for anthocyanin extraction regardless of the applied method. Considering extraction methods, for HVED, the highest extraction yields of anthocyanins were achieved at a frequency of 50 Hz for 15 min and for UAE at 80 °C after 15 min (1221.28 and 953.91 µg/g of dw, respectively). However, the highest total anthocyanin content (1757. 32 µg/g of dw) compared to the other two methods was observed in the extract obtained with PEF-assisted extraction in methanol-based solvent after 100 pulses and 20 kV/cm. These results are in agreement with Zhou and Huang [14], who also noticed that increasing the electric field intensity up to 20 kV/cm leads to an increase in the content of anthocyanins in the extract. The most optimal conditions for the extraction of anthocyanins by PEF in their case were at an electric field intensity of 20 kV/cm, acidified (0.1% of hydrochloric acid) 60% ethanol, and a liquid to liquid ratio of 1:6 (mL/mL). Under these conditions, the recovery of anthocyanins was 223.13 mg/L [14]. An increase in electric field intensity enhances the potential difference between the inside and outside of the cell membrane, causing a rupture of cell walls and improvement in the dissolution rate of cells [14]. Other studies on the extraction of anthocyanins showed that electrical treatments (HVED or PEF) were more effective when compared with ultrasound [14,39,40]. The study conducted by Barba et al. [15] on the extraction of anthocyanins from grape pomace showed that HVED (40 kV) was more effective compared with PEF (13.3 kV/cm). However, they also showed that PEF treatment allowed the selective recovery of extracts with an amount of anthocyanins 22 and 55% higher than with UAE and HVED, respectively [15].

### 3.3. Extraction of Phenolic Acids

Phenolic acids (Table 3) were the second most abundant polyphenolic group of compounds detected in blueberry pomace extract. In this study, three phenolic acids were identified: chlorogenic acid, caffeic acid, and 4-hydroxycinnamic acid. Chlorogenic and caffeic acids have been previously detected in blueberries. However, rather than 4-hydroxycinnamic acid, other phenolic acids were marked as dominant such as p-hydroxybenzoic acid, vanillic acid, or p-hydroxybenzoic acid [41,42]. In this study, phenolic acids content ranged from 119.21.73 to 625.47 µg/g of dw, and chlorogenic acid was the most abundant one found in blueberry pomace extract. Three-way ANOVA analysis showed that all investigated factors (HVED—solvent, time, and frequency; PEF—solvent, electric field intensity, and number of pulses; UAE—solvent, temperature, and time) had a significant effect (*p* < 0.05) on the extraction of phenolic acids. The highest extraction yields of phenolic acids with HVED were achieved in ethanol-based solvent at the frequency of 50 Hz for 15 min (442.90 µg/g of dw), while UAE showed to be more efficient in methanol-based solvent after 15 min at 80 °C (561.26 µg/g of dw). However, the highest TPC (625.47 µg/g of dw) compared with the other two methods, was observed in the extract obtained with PEF-assisted extraction in ethanol-based solvent after 100 pulses and 20 kV/cm. Other studies also showed promising results in PEF-assisted extraction of phenolic acids from different food by-products. In a study dealing with polyphenols extraction from peach by-products, Redondo et al. [43] showed that PEF application resulted in an increase in chlorogenic acid (16.3 mg/100 g), coumaric acid (0.8 mg/100 g), and chlorogenic acid (9.8mg/100 g) compared to the amounts extracted without the application of PEF (4.5, 0.2, and 2.5 and mg/100 g, respectively). Furthermore, Martín-García et al. [44], studying PEF-assisted extraction of polyphenols from brewery by-products, showed improvement in the p-coumaric acid and ferulic acid recovery from brewers spent grain compared with the conventional method.

### 3.4. Extraction of Flavanols

The third most abundant polyphenolic group of compounds was flavanols (Table 4). In this study, four flavanols were identified: catechin, epicatechin procyanidin B1, and procyanidin B2. Catechin, epicatechin, and only B-type procyanidins were previously identified in cultivated and wild diploid blueberry species by Wang et al. [45]. In this study, flavanols content in blueberry pomace extract ranged from 53.34 to 297.86 µg/g of dw and the most represented flavanol was procyanidin B2. Three-way ANOVA analysis showed that all investigated factors (HVED—solvent, time, and frequency; PEF—solvent, electric field intensity, and number of pulses; UAE—solvent, temperature, and time) had a significant effect (*p* < 0.05) on the extraction of flavanols. As for anthocyanins, methanol-based solvent proved as a better extraction solvent for flavanols extraction, regardless of the applied method. The highest total phenolic acid content (297.86 µg/g of dw) was observed in the extract obtained with PEF-assisted extraction in methanol-based solvent after 100 pulses and 20 kV/cm. For HVED, the highest extraction yields were achieved at the frequency of 50 Hz for 15 min and for UAE, at 80 °C for 15 min (182.47 and 156.04 µg/g of dw, respectively). Boussetta et al. [46] studied the HVED-assisted extraction of flavanols from grape pomace, showing an increase in the amount of extracted catechin and epicatechin. They also observed a negative effect of HVED at higher energy inputs (>80 kJ/kg) corresponding to the decrease in polyphenols content. In our study, the highest flavanol content was achieved at 22.27 kJ/kg; higher energy input (42.54 kJ/kg) resulted in a lower amount of flavanols. Interestingly, the highest amount of extracted flavanols was achieved with PEF-assisted extraction at 41.03 kJ/kg. This could be explained by the difference in the extraction mechanism of these two methods, where the HVED mechanism, among others, includes the generation of ozone, atomic hydrogen, and hydroxyl radicals during water photodissociation. The resulting oxidative chemical reactions could negatively affect the extracted polyphenols causing their damage. For example, formed ozone could react with water molecules to form hydrogen peroxide, which then decomposes to form hydroxyl radicals [46]. In particular, for high energy input, the quantity of produced oxidising species is increased and that may oxidise polyphenols. Furthermore, in the case of ethanol-based solvent, this can lead to the oxidation of ethanol in ethanal and in the formation of a flavanol–anthocyanin adduct with or without CH_3_−CH bridges originating from ethanal [47].

### 3.5. Extraction of Flavonols

The least abundant polyphenolic group of compounds found in the extract was flavonols (Table 5), among which myricetin was the most represented flavonol. Four flavonols were detected in blueberry pomace extract: myricetin, quercetin, kaempferol, and quercetin derivatives. These flavonols have been previously detected in blueberries [48]. In this study, flavonols content ranged from 44.53 to 157.54 µg/g of dw. Three-way ANOVA analysis showed that all investigated factors (HVED—solvent, time, and frequency; PEF—solvent, electric field intensity, and number of pulses; UAE—solvent, temperature, and time) had a significant effect (*p* < 0.05) on the extraction of flavonols. The highest total flavonols content (157.54 µg/g of dw) was observed in the extract obtained with PEF-assisted extraction in ethanol-based solvent after 100 pulses and 20 kV/cm. Bansal et al. [49] also showed that PEF extraction of quercetin from *Emblica Officinalis* is an effective treatment for the extraction of flavonols compared to conventional methods. Furthermore, Teherani et al. [50] proposed PEF (4.1 kV) as a very effective method for extraction of quercetin from onion by-products. Concerning the other two methods, higher extraction yields of flavonols with HVED were achieved in ethanol-based solvent at the frequency of 50 Hz for 15 min, while US-assisted extraction showed to be more efficient in methanol-based solvent after 15 min at 80 °C (121.90 and 98.63 µg/g of dw, respectively).

This has been shown also in other studies, which found that flavonols dissolve better in ethanol–water mixtures compared to methanol–water mixtures [51]. Numerous studies have found a significant effect of solvent type and extraction method on polyphenol yield from certain plant materials. The type of extraction solvent as well as the extraction method have a significant impact on the extraction yield of individual polyphenols from each individual plant material. There are some reports concerning optimisation of extraction conditions of phenolic compound content and antioxidant activities of some plant foods but some research works indicated the optimal procedure to usually be different for different plant matrices and different extraction methods [52,53].

Previous research has also shown that electrotechnologies (PEF or HVED) promote polyphenol extraction and that better yields can be achieved compared to other methods, including the US-assisted extraction method [14]. When applying PEF or HVED for the enhancement of the extraction of polyphenols from various raw materials, the main operating parameter is the treatment’s energy input [54]. In the present study, it was shown that HVED-assisted extraction had better extraction yield at 22.27 kJ/kg. Increasing energy input results in a decrease in extraction yield. As we already mentioned, the HVED mechanism of action includes generation of hydroxyl radicals during water photodissociation, atomic hydrogen and ozone, and for high energy input, the quantity of produced oxidising species is increased, which may cause oxidation of polyphenols. This may be the reason for obtaining a lower extraction yield compared to PEF-assisted extraction.

Higher extraction yields using PEF-assisted extraction at higher energy inputs (41.03 kJ/kg) can also be explained by a different mechanism of action of PEF, followed by which is the increasing diffusivity of the intracellular substances caused by permeabilisation of cell membranes, which ultimately improve extraction efficiency by increased mass transfer rate [55]. This process increases the extraction rates and yields of different active ingredients without affecting the quality of the extracted products [14]. Furthermore, higher extraction yields may be attributed to the ability of PEF to selectively induce the extraction of specific compounds. In fact, it has been reported that the specific group of polyphenols, their relative location in the plant tissue, their bounding capacity to the plant matrix, and their chemical structure and stability during processing at different conditions [15,28] might be crucial in obtaining extracts with specific biochemical profiles.

## 4. Conclusions

The results of this study have demonstrated that the extraction yield of phenolic compounds from blueberry pomace using HVED, PEF, and UAE is dependent on all investigated factors (HVED—solvent, time, and frequency; PEF—solvent, electric field intensity, and number of pulses; UAE—solvent, temperature, and time). Comparing the different extraction methods, it can be observed that both TPC and AA of extracts obtained by PEF were higher than those obtained employing HVED and UAE. Eighteen polyphenols have been positively identified by LC-MS/MS and quantified by HPLC-DAD. The highest anthocyanin (1757.32 µg/g of dw) and flavanol (297.86 µg/g of dw) yields were obtained in methanol-based solvent, while the highest phenolic acid (625.47 µg/g of dw) and flavonol (157.54 µg/g of dw) yields were obtained in ethanol-based solvent by PEF-assisted extraction at the energy input of 41.03 kJ/kg. These results indicated that PEF can improve the extraction yield of polyphenols from blueberry pomace and that it is a promising green extraction method.

## Figures and Tables

**Table 1:** Polyphenolic compounds and mass spectrometry variables found in blueberry pomace.

Phenolic Compound	Structure	Molecular Formula	[M − H]^−^ (*m*/*z*) ^a^	MS/MS (*m*/*z*) ^b^	Collision Energy (eV)	Rt (min) HPLC ^c^
Catechin	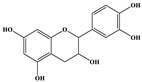	C_15_H_14_O_6_	289.006	245.020 203.115	17 22	9.53
Procyanidin B1	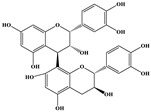	C_30_H_26_O_12_	577.033	407.066 288.931 424.977	26 25 26	10.07
Caffeic Acid	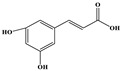	C_9_H_8_O_4_	178.978	135.033 134.010	19 28	10.66
Procyanidin B2	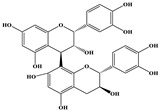	C_30_H_26_O_12_	577.033	407.066 288.931 424.977	26 25 26	11.58
Chlorogenic Acid	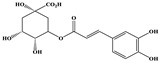	C_16_H_18_O_9_	353.000	191.074 85.090 93.073	22 43 45	12.38
Epicatechin	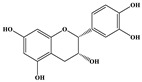	C_15_H_14_O_6_	289.006	245.020 203.115	17 22	13.79
4-Hydroxcinnamic acid	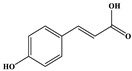	C_9_H_8_O_3_	163.016	119.072 163.016 163.016	18 37 38	14.70
Myricetin ^+^	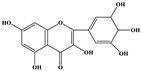	C_15_H_10_O_8_	319.000	153.027 217.062 245.062	31 31 27	19.00
Quercetin ^+^	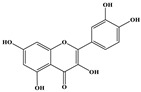	C_15_H_10_O_7_	303.098	229.106 153.046	28 33	19.86
Kaempferol	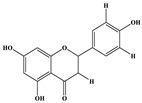	C_15_H_10_O_6_	285.078	184.919 239.126	30 35	20.97
Anthocyanins *						
Del-3-glu ^+^	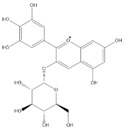	C_21_H_21_O_12_	465.1027			13.39
Del-3-ara ^+^	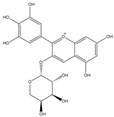	C_20_H_19_O_11_	435.0922			14.47
Pet-3-glu ^+^	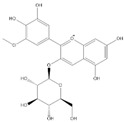	C_22_H_23_O_12_	479.1184			15.24
Cya-3-ara ^+^	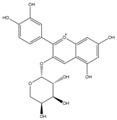	C_20_H_19_O_10_	419.0928			16.37
Peo-3-glu ^+^	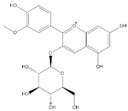	C_22_H_23_O_11_	463.1235			16.61
Peo-3-ara ^+^	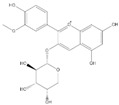	C_21_H_21_O_11_	433.1129			17.18
Mal-3-glu ^+^	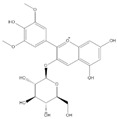	C_23_H_25_O_12_	493.1340			17.68

^a^ [M − H]^−^ = Molecular ion weight. ^b^ MS/MS = Fragmented anthocyanidin molecular weight. ^c^ -Rt = retention time (minutes) from the Jasco LC Net II high-performance liquid chromatography (HPLC). ^+^ Compound detected in positive mode [M + H]^+^. * Tentatively identified. Del-3-glu—delphinidin 3-glucoside; Del-3-ara—delphinidin-3-arabinoside; Pet-3-glu—petunidin 3-glucoside; Cya-3-ara—cyanidin-3-arabinoside; Peo-3-glu—peonidin 3-glucoside; Peo-3-ara—peonidin 3-arabinoside; Mal-3-glu—malvidin 3-glucoside.

**Table 2:** The anthocyanins content (µg/g of dw) in extracts obtained after high voltage electrical discharges (HVED), pulsed electric field (PEF), and ultrasound-assisted extraction (UAE) of blueberry pomace.

Ethanol		Anthocyanins							
HVED (Hz)	Minutes	Del-3-Glu	Del-3-ara	Pet-3-glu	Cya-3-ara	Peo-3-glu	Peo-3-ara	Mal-3-glu	Total
20	5	122.23 ± 3.08	87.77 ± 2.33	171.12 ± 4.91	44.73 ± 1.71	192.38 ± 5.3	7.14 ± 0.28	78.11 ± 2.69	703.48
20	10	157.04 ± 2.05	110.96 ± 1.34	188.35 ± 3.54	39.05 ± 1.14	228.56 ± 2.92	8.42 ± 0.16	100.74 ± 1.33	833.12
20	15	174.12 ± 4.22	124.02 ± 3.16	212.94 ± 4.97	44.99 ± 1.19	255.62 ± 5.96	9.36 ± 0.29	112.22 ± 2.84	933.27
50	5	146.66 ± 3.36	105.27 ± 2.44	178.60 ± 4.81	35.80 ± 0.89	213.30 ± 5.89	7.96 ± 0.3	93.88 ± 3.51	781.47
50	10	185.50 ± 2.90	133.14 ± 2.46	226.25 ± 4.94	43.09 ± 1.13	269.41 ± 3.89	10.49 ± 0.29	120.33 ± 2.3	988.21
50	15	102.24 ± 2.32	74.03 ± 1.81	128.17 ± 3.22	26.16 ± 0.81	152.06 ± 4.06	1.00 ± 0.02	67.03 ± 1.34	550.69
100	5	133.76 ± 3.86	95.75 ± 2.55	171.07 ± 11.34	37.44 ± 7.16	196.93 ± 10.19	7.65 ± 0.26	83.75 ± 2.54	726.35
100	10	178.95 ± 5.66	126.32 ± 3.61	222.76 ± 5.90	46.02 ± 1.57	259.50 ± 8.23	10.35 ± 0.26	113.66 ± 3.67	957.56
100	15	203.96 ± 5.67	144.19 ± 3.30	253.84 ± 5.37	52.92 ± 1.94	299.82 ± 8.56	11.93 ± 0.43	120.52 ± 3.93	1087.18
PEF (kV/cm)	Pulse No.								
10	10	185.94 ± 13.92	132.99 ± 3.71	225 ± 8.04	48.20 ± 1.14	298.26 ± 10.54	5.47 ± 0.07	127.01 ± 4.18	1022.87
10	50	187.96 ± 5.08	135.52 ± 3.1	241.02 ± 4.53	50.98 ± 2.60	316.21 ± 7.63	5.23 ± 0.14	131.25 ± 3.72	1068.17
10	100	190.89 ± 5.23	143.61 ± 3.97	256.63 ± 5.52	55.03 ± 2.34	348.97 ± 7.09	6.70 ± 0.24	147.45 ± 3.67	1149.28
15	10	175.75 ± 8.54	127.31 ± 6.84	217.25 ± 8.19	47.09 ± 1.66	279.67 ± 11.45	4.95 ± 0.23	117.26 ± 7.54	969.28
15	50	169.08 ± 2.75	126.12 ± 1.82	222.3 ± 4.18	47.60 ± 1.00	293.73 ± 4.69	4.69 ± 0.24	126.44 ± 2.05	989.96
15	100	193.49 ± 9.05	153.4 ± 9.19	289.65 ± 14.8	60.66 ± 2.96	400.71 ± 17.97	7.07 ± 0.29	169.94 ± 7.39	1274.92
20	10	209.32 ± 8.70	152.16 ± 6.09	262.42 ± 8.91	55.95 ± 2.77	340.43 ± 13.96	5.92 ± 0.27	145.56 ± 6.77	1171.76
20	50	220.56 ± 7.80	162.98 ± 4.34	301.2 ± 23.05	67.17 ± 15.76	386.54 ± 19.83	7.36 ± 0.16	162.10 ± 5.93	1307.91
20	100	279.39 ± 10.73	205.24 ± 7.90	370.58 ± 14.00	79.13 ± 2.9	477.61 ± 17.45	11.33 ± 0.51	201.26 ± 8.81	1624.54
US (T °C)	Minutes								
20	5	52.95 ± 0.87	40.80 ± 1.05	64.36 ± 1.22	12.40 ± 0.26	80.07 ± 2.45	1.40 ± 0.02	33.75 ± 0.95	285.73
20	10	76.36 ± 2.23	56.41 ± 1.76	94.01 ± 3.01	18.59 ± 0.61	114.16 ± 3.89	2.28 ± 0.06	49.33 ± 2.05	411.14
20	15	100.01 ± 5.53	72.44 ± 3.93	122.48 ± 6.23	23.44 ± 0.85	146.78 ± 7.75	4.12 ± 0.28	63.90 ± 3.98	533.17
40	5	62.29 ± 2.27	46.44 ± 1.87	73.13 ± 3.27	13.45 ± 0.36	82.88 ± 4.75	1.41 ± 0.04	37.10 ± 2.45	316.70
40	10	79.30 ± 3.16	58.12 ± 2.57	93.87 ± 4.98	17.38 ± 0.58	105.80 ± 5.8	1.90 ± 0.06	47.52 ± 2.61	403.89
40	15	120.50 ± 2.84	88.06 ± 2.19	153.57 ± 12.24	32.90 ± 8.39	181.80 ± 10.59	5.08 ± 0.13	77.01 ± 2.09	658.92
80	5	124.94 ± 2.59	92.61 ± 2.16	155.35 ± 3.96	29.97 ± 0.88	201.58 ± 4.94	4.13 ± 0.11	86.36 ± 1.95	694.94
80	10	146.36 ± 3.28	107.94 ± 2.15	183.81 ± 4.09	36.57 ± 0.65	226.42 ± 4.54	4.80 ± 0.10	98.60 ± 2.56	804.50
80	15	182.33 ± 6.15	131.75 ± 4.68	204.11 ± 7.91	43.37 ± 1.36	264.54 ± 9.86	6.29 ± 0.31	116.89 ± 4.68	949.28
Methanol									
HVED (Hz)	Minutes								
20	5	183.81 ± 4.19	128.97 ± 3.40	228.02 ± 6.46	46.92 ± 1.45	265.21 ± 7.32	10.79 ± 0.17	114.86 ± 2.85	978.58
20	10	205.46 ± 10.31	144.44 ± 7.08	254.27 ± 13.45	53.32 ± 2.80	299.05 ± 15.64	10.54 ± 0.53	131.10 ± 7.07	1098.18
20	15	222.23 ± 6.57	155.98 ± 4.94	275.22 ± 7.94	56.88 ± 2.42	325.47 ± 9.84	10.30 ± 0.61	143.05 ± 4.92	1189.13
50	5	187.32 ± 2.61	130.42 ± 2.15	231.34 ± 3.76	47.70 ± 0.90	271.26 ± 3.86	11.38 ± 0.79	116.95 ± 1.18	996.37
50	10	217.38 ± 14.17	152.38 ± 10.08	268.88 ± 18.97	55.38 ± 4.23	319.06 ± 21.7	11.43 ± 0.36	139.19 ± 9.52	1163.70
50	15	228.11 ± 5.61	158.83 ± 3.48	283.99 ± 6.87	58.86 ± 1.62	334.39 ± 8.52	11.31 ± 0.25	145.79 ± 3.91	1221.28
100	5	167.92 ± 3.18	117.15 ± 1.96	205.56 ± 3.39	40.96 ± 1.22	241.71 ± 4.65	8.86 ± 0.35	105.92 ± 2.10	888.08
100	10	201.16 ± 7.87	139.64 ± 5.13	247.67 ± 9.74	52.06 ± 1.80	291.32 ± 10.85	9.87 ± 0.52	127.33 ± 4.68	1069.05
100	15	204.98 ± 5.70	142.35 ± 4.02	253.36 ± 6.90	52.96 ± 1.90	298.10 ± 9.05	9.83 ± 0.22	130.07 ± 3.38	1091.65
PEF (kV/cm)	Pulse No.								
10	10	177.70 ± 10.78	125.50 ± 5.33	222.39 ± 12.60	48.79 ± 3.37	270.18 ± 14.44	1.41 ± 0.20	116.78 ± 8.72	962.75
10	50	202.38 ± 10.85	143.46 ± 11.05	255.74 ± 20.02	52.43 ± 4.92	320.43 ± 23.07	1.50 ± 0.17	136.94 ± 11.36	1112.88
10	100	206.96 ± 3.71	146.98 ± 1.52	263.92 ± 12.29	58.49 ± 3.05	341.00 ± 10.18	1.73 ± 0.19	148.07 ± 3.24	1167.15
15	10	210.21 ± 11.62	149.62 ± 9.02	265.86 ± 13.47	59.77 ± 3.31	332.22 ± 16.36	1.56 ± 0.39	141.98 ± 6.19	1161.22
15	50	239.46 ± 6.99	174.41 ± 8.29	295.52 ± 20.43	62.89 ± 5.79	347.15 ± 24.64	1.83 ± 0.19	155.87 ± 11.05	1277.13
15	100	248.33 ± 12.46	178.99 ± 10.51	326.64 ± 17.45	68.57 ± 4.26	419.18 ± 27.02	1.83 ± 0.18	180.08 ± 9.31	1423.62
20	10	228.41 ± 7.58	161.90 ± 7.95	294.41 ± 11.87	60.80 ± 2.34	367.03 ± 13.34	1.72 ± 0.06	156.65 ± 6.17	1270.92
20	50	263.36 ± 5.38	183.62 ± 3.98	343.53 ± 2.73	77.21 ± 7.35	472.62 ± 40.17	2.01 ± 0.18	189.68 ± 10.29	1532.03
20	100	325.13 ± 10.14	230.93 ± 7.35	408.30 ± 12.17	87.65 ± 2.80	485.47 ± 18.70	2.63 ± 0.09	217.21 ± 6.38	1757.32
US (T °C)	Minutes								
20	5	73.60 ± 3.27	53.96 ± 2.26	90.33 ± 4.12	18.59 ± 0.86	114.96 ± 5.35	0.66 ± 0.04	49.84 ± 2.37	401.94
20	10	76.36 ± 2.23	56.41 ± 1.76	94.01 ± 3.01	18.59 ± 0.61	114.16 ± 3.89	2.28 ± 0.06	49.33 ± 2.05	411.14
20	15	100.01 ± 5.53	72.44 ± 3.93	122.48 ± 6.23	23.44 ± 0.85	146.78 ± 7.75	4.12 ± 0.28	63.90 ± 3.98	533.17
40	5	102.24 ± 2.32	74.03 ± 1.81	128.17 ± 3.22	26.16 ± 0.81	152.06 ± 4.06	1.00 ± 0.02	67.03 ± 1.34	550.69
40	10	116.83 ± 5.12	84.49 ± 3.55	145.41 ± 6.57	29.93 ± 1.29	169.60 ± 7.37	1.12 ± 0.06	75.61 ± 3.64	622.99
40	15	132.09 ± 8.63	94.68 ± 6.02	164.35 ± 11.45	33.62 ± 1.85	191.19 ± 12.97	1.28 ± 0.12	85.19 ± 5.80	702.40
80	5	108.87 ± 4.58	79.96 ± 3.46	132.15 ± 6.63	28.00 ± 2.11	148.29 ± 8.72	0.89 ± 0.09	67.40 ± 3.76	565.56
80	10	156.31 ± 8.85	114.30 ± 6.40	190.59 ± 10.71	41.67 ± 2.64	214.49 ± 13.5	1.40 ± 0.12	97.52 ± 5.94	816.28
80	15	175.59 ± 7.32	128.90 ± 5.07	236.13 ± 7.99	45.28 ± 1.44	252.73 ± 10.43	2.16 ± 0.07	113.12 ± 4.67	953.91

Del-3-glu—delphinidin 3-glucoside; Del-3-ara—delphinidin-3-arabinoside; Pet-3-glu—petunidin 3-glucoside; Cya-3-ara—cyanidin-3-arabinoside; Peo-3-glu—peonidin 3-glucoside; Peo-3-ara—peonidin 3- arabinoside; Mal-3-glu—malvidin 3-glucoside.

**Table 3:** Phenolic acids content (µg/g of dw) in the analysed extracts obtained after high voltage electrical discharges (HVED), pulsed electric field (PEF), and ultrasound-assisted extraction (UAE) of blueberry pomace.

Ethanol		Phenolic Acids			
HVED (Hz)	Minutes	Chlorogenic Acid	Caffeic Acid	4-HCA	Total
20	5	224.30 ± 7.73	2.52 ± 0.06	16.82 ± 0.76	243.64
20	10	282.73 ± 5.57	2.91 ± 0.11	21.20 ± 0.21	306.84
20	15	322.65 ± 7.94	3.18 ± 0.12	23.29 ± 0.69	349.12
50	5	302.03 ± 5.67	2.98 ± 0.08	20.27 ± 0.37	325.28
50	10	373.83 ± 7.18	3.57 ± 0.10	24.59 ± 0.42	401.99
50	15	425.42 ± 14.87	1.63 ± 0.05	15.85 ± 0.31	442.90
100	5	256.38 ± 8.84	2.78 ± 0.07	18.53 ± 0.57	277.69
100	10	334.03 ± 11.74	3.32 ± 0.11	23.93 ± 0.48	361.28
100	15	378.60 ± 12.13	3.63 ± 0.10	26.67 ± 0.90	408.90
PEF (kV/cm)	Pulse No.				
10	10	297.91 ± 10.07	3.39 ± 0.08	30.43 ± 1.23	331.73
10	50	216.55 ± 5.45	3.70 ± 0.14	32.55 ± 1.06	252.80
10	100	279.64 ± 8.22	3.47 ± 0.12	33.43 ± 1.37	316.54
15	10	197.80 ± 10.44	3.46 ± 0.16	28.74 ± 0.81	230.00
15	50	188.52 ± 4.82	3.66 ± 0.11	29.89 ± 0.67	222.07
15	100	253.91 ± 11.81	4.99 ± 0.17	35.49 ± 1.73	294.39
20	10	270.83 ± 3.46	3.80 ± 0.24	33.90 ± 1.13	308.53
20	50	372.75 ± 15.78	4.36 ± 0.11	37.52 ± 1.29	414.63
20	Minutes	568.86 ± 4.83	6.86 ± 0.19	49.75 ± 1.51	625.47
UAE (T °C)	Minutes				
20	5	170.20 ± 5.84	1.37 ± 0.04	10.13 ± 0.10	181.70
20	10	197.97 ± 21.29	1.61 ± 0.05	13.22 ± 0.29	212.80
20	15	250.41 ± 4.87	1.77 ± 0.15	16.69 ± 0.21	268.87
40	5	143.46 ± 2.92	1.44 ± 0.05	10.66 ± 0.54	155.56
40	10	184.11 ± 9.56	1.66 ± 0.06	12.61 ± 0.29	198.38
40	15	442.98 ± 13.05	2.13 ± 0.06	18.40 ± 0.47	463.51
80	5	350.38 ± 33.72	2.25 ± 0.04	18.85 ± 0.38	371.48
80	10	451.21 ± 11.93	2.57 ± 0.34	19.35 ± 0.28	473.13
80	15	471.95 ± 2.17	2.69 ± 0.1	20.24 ± 0.85	494.88
Methanol					
HVED (Hz)					
20	5	168.27 ± 5.04	2.81 ± 0.06	23.79 ± 0.59	194.87
20	10	190.52 ± 11.7	2.68 ± 0.14	23.91 ± 0.23	217.11
20	15	210.23 ± 9.57	2.63 ± 0.16	26.02 ± 0.97	238.88
50	5	166.81 ± 2.19	2.68 ± 0.10	26.93 ± 1.70	196.42
50	10	197.98 ± 14.93	2.67 ± 0.16	27.74 ± 0.92	228.39
50	15	209.00 ± 7.29	2.67 ± 0.17	27.89 ± 0.61	239.56
100	5	142.83 ± 2.73	2.46 ± 0.09	21.62 ± 0.32	166.91
100	10	173.46 ± 8.48	2.37 ± 0.14	25.11 ± 0.80	200.94
100	15	179.62 ± 6.80	2.27 ± 0.17	25.59 ± 0.64	207.48
PEF (kV/cm)	Pulse No.				
10	10	130.87 ± 6.51	2.78 ± 0.20	26.88 ± 0.57	160.53
10	50	116.84 ± 10.36	3.37 ± 0.21	29.47 ± 2.23	149.68
10	100	85.53 ± 2.56	2.90 ± 0.11	30.78 ± 1.05	119.21
15	10	97.50 ± 4.74	3.55 ± 0.29	28.42 ± 1.44	129.47
15	50	126.95 ± 3.15	3.51 ± 0.24	32.55 ± 2.12	163.01
15	100	133.41 ± 5.44	3.95 ± 0.27	36.27 ± 2.71	173.63
20	10	97.88 ± 6.61	4.06 ± 0.14	32.43 ± 1.43	134.37
20	50	157.15 ± 14.03	4.30 ± 0.42	40.30 ± 2.97	201.75
20	100	551.29 ± 5.08	4.93 ± 0.21	44.57 ± 1.43	600.79
UAE (T °C)	Minutes				
20	5	220.06 ± 15.75	1.31 ± 0.11	12.10 ± 0.42	233.47
20	10	342.73 ± 6.23	1.61 ± 0.05	13.22 ± 0.29	357.56
20	15	439.57 ± 29.82	1.77 ± 0.15	16.69 ± 0.21	458.03
40	5	425.42 ± 14.87	1.63 ± 0.05	15.85 ± 0.31	442.90
40	10	461.50 ± 14.64	1.77 ± 0.07	17.76 ± 0.74	481.03
40	15	539.92 ± 20.67	1.93 ± 0.08	19.41 ± 1.10	561.26
80	5	227.08 ± 11.94	1.79 ± 0.06	16.86 ± 0.69	245.73
80	10	332.86 ± 20.71	2.19 ± 0.10	22.58 ± 1.35	357.63
80	15	372.99 ± 14.32	2.32 ± 0.07	26.24 ± 0.86	401.55

4-HCA—4 hydroxycinnamic acid.

**Table 4:** Flavanols content (µg/g of dw) in the analysed extracts obtained after high voltage electrical discharges (HVED), pulsed electric field (PEF), and ultrasound-assisted extraction (UAE) of blueberry pomace.

Ethanol		Flavanols				
HVED (Hz)	Minutes	Catechin	PCB1	PCB2	Epicatechin	Total
20	5	18.77 ± 1.09	5.21 ± 0.16	44.13 ± 0.67	31.13 ± 0.70	99.24
20	10	21.63 ± 0.90	5.40 ± 0.26	54.50 ± 0.75	35.22 ± 2.10	116.75
20	15	26.19 ± 0.85	5.56 ± 0.20	61.00 ± 2.03	42.37 ± 1.64	135.12
50	5	23.38 ± 0.76	5.37 ± 0.07	56.75 ± 0.77	35.61 ± 1.33	121.11
50	10	27.38 ± 0.51	5.69 ± 0.15	68.75 ± 1.30	44.52 ± 0.87	146.34
50	15	20.00 ± 0.70	7.20 ± 0.18	37.71 ± 1.08	30.91 ± 0.77	95.82
100	5	21.70 ± 0.42	5.27 ± 0.05	49.59 ± 1.97	35.65 ± 0.91	112.21
100	10	28.16 ± 0.24	5.67 ± 0.10	64.23 ± 2.17	48.65 ± 0.79	146.71
100	15	30.39 ± 0.74	5.91 ± 0.13	72.98 ± 2.49	53.95 ± 1.18	163.23
PEF (kV/cm)	Pulse No.					
10	10	39.56 ± 0.81	5.98 ± 0.11	80.32 ± 3.30	54.43 ± 12.67	180.29
10	50	46.09 ± 0.38	5.83 ± 0.21	85.74 ± 1.13	45.54 ± 1.62	183.20
10	100	46.02 ± 0.88	5.99 ± 0.32	90.81 ± 3.21	50.74 ± 2.92	193.56
15	10	37.74 ± 0.90	5.69 ± 0.27	72.01 ± 3.69	44.35 ± 0.88	159.79
15	50	43.14 ± 1.86	5.90 ± 0.08	80.86 ± 3.37	40.47 ± 4.31	170.37
15	100	57.46 ± 1.76	6.28 ± 0.13	111.91 ± 5.26	47.91 ± 1.99	223.56
20	10	45.03 ± 1.86	6.14 ± 0.19	86.20 ± 3.91	47.86 ± 0.43	185.23
20	50	49.84 ± 1.45	6.43 ± 0.15	94.64 ± 2.93	51.20 ± 1.55	202.11
20	100	59.34 ± 2.54	7.65 ± 0.17	123.16 ± 4.58	74.72 ± 1.55	264.87
UAE (T °C)	Minutes					
20	5	14.30 ± 0.30	4.75 ± 0.16	26.02 ± 1.60	14.08 ± 0.42	59.15
20	10	15.98 ± 0.38	5.21 ± 0.10	30.32 ± 1.07	18.87 ± 0.39	70.38
20	15	18.19 ± 1.84	5.36 ± 0.39	36.73 ± 2.99	23.48 ± 1.25	83.76
40	5	12.41 ± 0.32	4.97 ± 0.13	22.21 ± 0.83	13.75 ± 1.02	53.34
40	10	15.09 ± 0.35	5.26 ± 0.11	26.99 ± 0.83	17.99 ± 0.88	65.33
40	15	22.79 ± 0.73	5.90 ± 0.14	45.30 ± 1.50	26.55 ± 1.57	100.54
80	5	25.67 ± 0.87	6.08 ± 0.13	54.39 ± 1.10	26.30 ± 1.66	112.44
80	10	28.04 ± 1.70	5.79 ± 0.10	57.73 ± 2.48	29.48 ± 2.53	121.04
80	15	30.34 ± 1.66	5.70 ± 0.25	63.08 ± 1.68	34.84 ± 2.37	133.96
Methanol						
HVED (Hz)	Minutes					
20	5	26.31 ± 0.25	8.45 ± 0.19	64.38 ± 1.86	53.91 ± 0.83	153.05
20	10	28.43 ± 0.43	7.12 ± 0.65	62.25 ± 1.47	55.09 ± 0.70	152.89
20	15	30.96 ± 1.28	6.13 ± 0.55	68.87 ± 4.43	59.55 ± 2.82	165.51
50	5	32.48 ± 1.91	6.29 ± 0.46	73.07 ± 4.55	64.02 ± 3.79	175.86
50	10	33.12 ± 0.94	6.24 ± 0.40	76.06 ± 2.75	64.94 ± 1.45	180.36
50	15	34.02 ± 0.97	6.23 ± 0.56	76.34 ± 2.31	65.88 ± 1.58	182.47
100	5	25.48 ± 0.19	6.42 ± 0.38	53.89 ± 1.08	49.55 ± 1.22	135.34
100	10	29.62 ± 1.10	5.49 ± 0.31	64.01 ± 3.05	58.00 ± 2.02	157.12
100	15	30.57 ± 0.83	5.53 ± 0.35	66.73 ± 2.08	59.11 ± 1.59	161.94
PEF (kV/cm)	Pulse No.					
10	10	33.63 ± 1.64	7.68 ± 0.36	68.18 ± 3.76	56.48 ± 3.01	165.97
10	50	44.86 ± 2.38	8.74 ± 0.67	86.01 ± 5.61	60.54 ± 4.57	200.15
10	100	45.21 ± 0.34	8.04 ± 0.32	89.39 ± 2.71	63.01 ± 1.46	205.65
15	10	44.53 ± 1.98	8.63 ± 0.84	86.5 ± 3.69	65.15 ± 2.35	204.81
15	50	50.42 ± 2.96	9.47 ± 0.64	94.44 ± 6.92	70.83 ± 5.47	225.16
15	100	57.35 ± 2.54	10.00 ± 0.26	110.84 ± 7.62	75.88 ± 5.03	254.07
20	10	49.49 ± 2.08	8.52 ± 0.33	92.4 ± 5.37	70.75 ± 5.65	221.16
20	50	60.57 ± 3.81	10.74 ± 0.09	119.25 ± 8.51	85.50 ± 7.39	276.06
20	100	59.27 ± 1.40	12.11 ± 0.44	130.3 ± 4.81	96.18 ± 3.39	297.86
UAE (T °C)	Minutes					
20	5	18.11 ± 0.89	6.11 ± 0.71	32.79 ± 2.84	22.46 ± 1.42	79.47
20	10	15.98 ± 0.38	5.21 ± 0.10	30.32 ± 1.07	18.87 ± 0.39	70.38
20	15	18.19 ± 1.84	5.36 ± 0.39	36.73 ± 2.99	23.48 ± 1.25	83.76
40	5	20.00 ± 0.70	7.20 ± 0.18	37.71 ± 1.08	30.91 ± 0.77	95.82
40	10	21.77 ± 0.84	7.94 ± 0.23	41.79 ± 1.83	35.62 ± 1.44	107.12
40	15	23.30 ± 1.30	8.42 ± 0.29	45.58 ± 2.83	39.64 ± 2.51	116.94
80	5	19.58 ± 0.82	7.62 ± 0.11	37.86 ± 1.93	33.08 ± 1.36	98.14
80	10	25.65 ± 1.30	9.13 ± 0.33	52.00 ± 2.74	46.51 ± 3.05	133.29
80	15	30.98 ± 1.06	9.66 ± 0.31	63.05 ± 2.38	52.35 ± 2.08	156.04

PCB1—procyanidin B1; PCB2—procyanidin B2.

**Table 5:** Flavonols content (µg/g of dw) in the analysed extracts obtained after high voltage electrical discharges (HVED), pulsed electric field (PEF), and ultrasound-assisted extraction (UAE) of blueberry pomace.

Ethanol		Flavonols				
HVED (Hz)	Minutes	Myricetin	Que-der	Quercetin	Kaempferol	Total
20	5	39.08 ± 0.88	9.66 ± 0.20	8.57 ± 0.14	13.30 ± 0.33	70.61
20	10	49.10 ± 0.61	10.54 ± 0.40	9.54 ± 0.24	15.95 ± 0.20	85.13
20	15	55.08 ± 1.52	10.78 ± 0.27	9.75 ± 0.26	17.12 ± 0.38	92.73
50	5	48.18 ± 1.16	10.66 ± 0.26	9.50 ± 0.20	15.44 ± 0.27	83.78
50	10	59.87 ± 1.34	12.31 ± 0.23	10.72 ± 0.17	17.88 ± 0.30	100.78
50	15	76.89 ± 0.77	13.22 ± 0.16	9.41 ± 0.17	12.38 ± 0.28	121.90
100	5	42.90 ± 1.30	10.29 ± 0.20	9.05 ± 0.15	14.18 ± 0.27	76.42
100	10	56.06 ± 1.96	12.10 ± 0.27	10.39 ± 0.24	17.30 ± 0.51	95.85
100	15	63.97 ± 2.25	13.05 ± 0.28	11.18 ± 0.23	19.15 ± 0.50	107.35
PEF (kV/cm)	Pulse No.					
10	10	54.30 ± 1.89	14.00 ± 0.26	14.75 ± 0.36	25.61 ± 0.70	108.66
10	50	59.51 ± 1.86	13.87 ± 0.54	14.93 ± 0.33	26.18 ± 1.02	114.49
10	100	62.48 ± 2.06	15.14 ± 0.41	15.59 ± 0.40	28.37 ± 0.95	121.58
15	10	51.94 ± 3.06	12.46 ± 0.47	14.24 ± 0.63	22.64 ± 1.20	101.28
15	50	58.82 ± 1.76	13.52 ± 0.56	14.76 ± 0.50	25.50 ± 0.76	112.60
15	100	72.40 ± 3.52	16.47 ± 0.52	16.95 ± 0.57	32.21 ± 1.55	138.03
20	10	61.91 ± 3.42	13.91 ± 0.37	15.96 ± 0.58	26.32 ± 1.18	118.10
20	50	67.86 ± 2.32	15.25 ± 0.30	16.91 ± 0.39	30.32 ± 0.85	130.34
20	100	84.90 ± 3.56	17.39 ± 0.55	20.17 ± 0.70	35.08 ± 1.24	157.54
UAE (T °C)	Minutes					
20	5	20.00 ± 0.84	7.74 ± 0.16	7.98 ± 0.14	10.08 ± 0.28	45.80
20	10	24.31 ± 0.89	8.50 ± 0.16	8.84 ± 0.17	11.39 ± 0.32	53.04
20	15	29.22 ± 1.74	9.52 ± 0.31	10.09 ± 0.39	13.23 ± 0.60	62.06
40	5	19.38 ± 1.00	7.54 ± 0.24	8.04 ± 0.25	9.57 ± 0.31	44.53
40	10	22.76 ± 1.09	8.14 ± 0.22	8.72 ± 0.19	10.67 ± 0.40	50.29
40	15	34.76 ± 0.98	10.34 ± 0.17	11.19 ± 0.22	14.74 ± 0.31	71.03
80	5	40.36 ± 0.90	11.15 ± 0.15	11.87 ± 0.18	16.11 ± 0.28	79.49
80	10	43.50 ± 1.07	11.16 ± 0.19	12.59 ± 0.24	16.74 ± 0.37	83.99
80	15	49.87 ± 1.94	11.38 ± 0.39	13.94 ± 0.45	18.11 ± 0.60	93.30
Methanol						
HVED (Hz)	Minutes					
20	5	38.36 ± 1.12	11.63 ± 0.17	8.71 ± 0.13	16.17 ± 0.35	74.87
20	10	38.24 ± 0.31	11.28 ± 0.43	8.82 ± 0.06	16.10 ± 0.33	74.44
20	15	42.76 ± 2.67	9.90 ± 0.46	8.68 ± 0.29	16.52 ± 0.79	77.86
50	5	45.80 ± 3.40	10.80 ± 0.57	9.18 ± 0.43	17.51 ± 1.08	83.29
50	10	47.69 ± 2.46	11.31 ± 0.42	9.44 ± 0.23	18.21 ± 0.65	86.65
50	15	48.53 ± 1.85	11.07 ± 0.35	9.47 ± 0.29	18.56 ± 0.61	87.63
100	5	34.71 ± 0.61	9.86 ± 0.30	8.34 ± 0.12	14.46 ± 0.24	67.37
100	10	40.67 ± 1.52	9.09 ± 0.52	8.32 ± 0.30	15.53 ± 0.67	73.61
100	15	43.01 ± 1.59	9.39 ± 0.51	8.47 ± 0.28	16.23 ± 0.48	77.10
PEF (kV/cm)	Pulse No.					
10	10	43.27 ± 2.33	11.72 ± 0.42	12.33 ± 0.42	20.74 ± 1.08	88.06
10	50	52.92 ± 4.42	13.45 ± 0.67	13.48 ± 1.05	25.11 ± 1.67	104.96
10	100	54.94 ± 1.32	14.12 ± 0.42	13.23 ± 0.22	25.93 ± 0.61	108.22
15	10	52.30 ± 2.56	13.13 ± 0.34	14.15 ± 0.27	24.54 ± 1.35	104.12
15	50	61.18 ± 4.42	14.73 ± 0.83	14.93 ± 0.61	27.93 ± 1.21	118.77
15	100	66.01 ± 3.94	14.55 ± 0.79	15.13 ± 0.99	29.05 ± 1.81	124.74
20	10	57.90 ± 3.30	12.64 ± 0.50	15.01 ± 0.87	25.09 ± 1.19	110.64
20	50	72.78 ± 6.29	15.83 ± 1.09	16.29 ± 0.75	32.13 ± 2.45	137.03
20	100	83.21 ± 3.17	15.48 ± 0.65	18.45 ± 0.58	33.26 ± 1.23	150.40
UAE (T °C)	Minutes					
20	5	21.42 ± 1.00	8.20 ± 0.19	8.35 ± 0.22	10.89 ± 0.33	48.86
20	10	24.31 ± 0.89	8.50 ± 0.16	8.84 ± 0.17	11.39 ± 0.32	53.04
20	15	29.22 ± 1.74	9.52 ± 0.31	10.09 ± 0.39	13.23 ± 0.60	62.06
40	5	26.89 ± 0.77	9.22 ± 0.16	9.41 ± 0.17	12.38 ± 0.28	57.90
40	10	29.81 ± 1.44	9.73 ± 0.28	10.05 ± 0.27	13.29 ± 0.48	62.88
40	15	33.20 ± 2.15	10.42 ± 0.39	10.75 ± 0.44	14.53 ± 0.70	68.90
80	5	27.98 ± 1.38	9.15 ± 0.26	9.53 ± 0.30	12.11 ± 0.45	58.77
80	10	38.78 ± 2.40	10.89 ± 0.38	11.76 ± 0.50	15.04 ± 0.73	76.47
80	15	56.11 ± 1.82	12.11 ± 0.29	13.18 ± 0.39	17.23 ± 0.51	98.63

Que-der—Quercetin derivate.

## References

[1] Skrovankova S., Sumczynski D., Mlcek J., Jurikova T., Sochor J. (2015). Bioactive compounds and antioxidant activity in different types of berries. Int. J. Mol. Sci..

[2] Michalska A., Łysiak G. (2015). Bioactive compounds of blueberries: Post-harvest factors influencing the nutritional value of products. Int. J. Mol. Sci..

[3] Tagliani C., Perez C., Curutchet A., Arcia P., Cozzano S. (2019). Blueberry pomace, valorization of an industry by-product source of fibre with antioxidant capacity. Food Sci. Technol..

[4] Bamba B.S.B., Shi J., Tranchant C.C., Xue S.J., Forney C.F., Lim L.T. (2018). Influence of extraction conditions on ultrasound-assisted recovery of bioactive phenolics from blueberry pomace and their antioxidant activity. Molecules.

[5] Abdulkhaleq L.A., Assi M.A., Noor M.H.M., Abdullah R., Saad M.Z., Taufiq-Yap Y.H. (2017). Therapeutic uses of epicatechin in diabetes and cancer. Vet. World.

[6] Gullón B., Lú-Chau T.A., Moreira M.T., Lema J.M., Eibes G. (2017). Rutin: A review on extraction, identification and purification methods, biological activities and approaches to enhance its bioavailability. Trends Food Sci. Technol..

[7] Naveed M., Hejazi V., Abbas M., Kamboh A.A., Khan G.J., Shumzaid M., Ahmad F., Babazadeh D., FangFang X., Modarresi-Ghazani F. (2018). Chlorogenic acid (CGA): A pharmacological review and call for further research. Biomed. Pharmacother..

[8] Rauf A., Imran M., Abu-Izneid T., Ul-Haq I., Patel S., Pan X., Naz S., Sanches Silva A., Saeed F., Rasul Suleria H.A. (2019). Proanthocyanidins: A comprehensive review. Biomed. Pharmacother..

[9] Šarić B., Mišan A., Mandić A., Nedeljković N., Pojić M., Pestorić M., Đilas S. (2016). Valorisation of raspberry and blueberry pomace through the formulation of value-added gluten-free cookies. J. Food Sci. Technol..

[10] Da Silva R.P.F.F., Rocha-Santos T.A.P., Duarte A.C. (2016). Supercritical fluid extraction of bioactive compounds. TrAC–Trends Anal. Chem..

[11] Armenta S., Garrigues S., de la Guardia M. (2015). The role of green extraction techniques in Green Analytical Chemistry. TrAC–Trends Anal. Chem..

[12] Galanakis C.M. (2013). Emerging technologies for the production of nutraceuticals from agricultural by-products: A viewpoint of opportunities and challenges. Food Bioprod. Process..

[13] Li Z., Fan Y., Xi J. (2019). Recent advances in high voltage electric discharge extraction of bioactive ingredients from plant materials. Food Chem..

[14] Zhou Y., Zhao X., Huang H. (2015). Effects of Pulsed Electric Fields on Anthocyanin Extraction Yield of Blueberry Processing By-Products. J. Food Process. Preserv..

[15] Barba F.J., Galanakis C.M., Esteve M.J., Frigola A., Vorobiev E. (2015). Potential use of pulsed electric technologies and ultrasounds to improve the recovery of high-added value compounds from blackberries. J. Food Eng..

[16] Chemat F., Vian M.A., Cravotto G. (2012). Green extraction of natural products: Concept and principles. Int. J. Mol. Sci..

[17] Brianceau S., Turk M., Vitrac X., Vorobiev E. (2016). High voltage electric discharges assisted extraction of phenolic compounds from grape stems: Effect of processing parameters on flavan-3-ols, flavonols and stilbenes recovery. Innov. Food Sci. Emerg. Technol..

[18] Boussetta N., Lebovka N., Vorobiev E., Adenier H., Bedel-Cloutour C., Lanoisellé J.L. (2009). Electrically assisted extraction of soluble matter from chardonnay grape skins for polyphenol recovery. J. Agric. Food Chem..

[19] Boussetta N., Lanoisellé J.L., Bedel-Cloutour C., Vorobiev E. (2009). Extraction of soluble matter from grape pomace by high voltage electrical discharges for polyphenol recovery: Effect of sulphur dioxide and thermal treatments. J. Food Eng..

[20] Xi J., He L., Yan L.G. (2017). Continuous extraction of phenolic compounds from pomegranate peel using high voltage electrical discharge. Food Chem..

[21] Yan L.G., Deng Y., Ju T., Wu K., Xi J. (2018). Continuous high voltage electrical discharge extraction of flavonoids from peanut shells based on “annular gap type” treatment chamber. Food Chem..

[22] Parniakov O., Barba F.J., Grimi N., Lebovka N., Vorobiev E. (2014). Impact of pulsed electric fields and high voltage electrical discharges on extraction of high-added value compounds from papaya peels. Food Res. Int..

[23] Puértolas E., Luengo E., Álvarez I., Raso J. (2012). Improving Mass Transfer to Soften Tissues by Pulsed Electric Fields: Fundamentals and Applications. Annu. Rev. Food Sci. Technol..

[24] Barba F.J., Grimi N., Vorobiev E. (2014). New Approaches for the Use of Non-conventional Cell Disruption Technologies to Extract Potential Food Additives and Nutraceuticals from Microalgae. Food Eng. Rev..

[25] Luengo E., Álvarez I., Raso J. (2013). Improving the pressing extraction of polyphenols of orange peel by pulsed electric fields. Innov. Food Sci. Emerg. Technol..

[26] Puértolas E., Cregenzán O., Luengo E., Álvarez I., Raso J. (2013). Pulsed-Electric-Field-Assisted extraction of anthocyanins from purple-fleshed potato. Food Chem..

[27] Boussetta N., Vorobiev E., Le L.H., Cordin-Falcimaigne A., Lanoisellé J.L. (2012). Application of electrical treatments in alcoholic solvent for polyphenols extraction from grape seeds. LWT–Food Sci. Technol..

[28] Corrales M., Toepfl S., Butz P., Knorr D., Tauscher B. (2008). Extraction of anthocyanins from grape by-products assisted by ultrasonics, high hydrostatic pressure or pulsed electric fields: A comparison. Innov. Food Sci. Emerg. Technol..

[29] Chemat F., Rombaut N., Sicaire A.G., Meullemiestre A., Fabiano-Tixier A.S., Abert-Vian M. (2017). Ultrasound assisted extraction of food and natural products. Mechanisms, techniques, combinations, protocols and applications. A review. Ultrason. Sonochem..

[30] Tiwari B.K. (2015). Ultrasound: A clean, green extraction technology. TrAC–Trends Anal. Chem..

[31] Wang T., Guo N., Wang S.X., Kou P., Zhao C.J., Fu Y.J. (2018). Ultrasound-Negative pressure cavitation extraction of phenolic compounds from blueberry leaves and evaluation of its DPPH radical scavenging activity. Food Bioprod. Process..

[32] Zhang H., Tchabo W., Ma Y. (2017). Quality of extracts from blueberry pomace by high hydrostatic pressure, ultrasonic, microwave and heating extraction: A comparison study. Emir. J. Food Agric..

[33] Singleton V.L., Rossi J.A. (1965). Colorimetry of Total Phenolics with Phosphomolybdic-Phosphotungstic Acid Reagents. Am. J. Enol. Vitic..

[34] Brand-Williams W., Cuvelier M.E., Berset C. (1995). Use of a free radical method to evaluate antioxidant activity. LWT–Food Sci. Technol..

[35] Barnes J.S., Nguyen H.P., Shen S., Schug K.A. (2009). General method for extraction of blueberry anthocyanins and identification using high performance liquid chromatography-electrospray ionization-ion trap-time of flight-mass spectrometry. J. Chromatogr. A.

[36] El Kantar S., Boussetta N., Lebovka N., Foucart F., Rajha H.N., Maroun R.G., Louka N., Vorobiev E. (2018). Pulsed electric field treatment of citrus fruits: Improvement of juice and polyphenols extraction. Innov. Food Sci. Emerg. Technol..

[37] Stein-Chisholm R., Beaulieu J., Grimm C., Lloyd S. (2017). LC–MS/MS and UPLC–UV Evaluation of Anthocyanins and Anthocyanidins during Rabbiteye Blueberry Juice Processing. Beverages.

[38] You Q., Wang B., Chen F., Huang Z., Wang X., Luo P.G. (2011). Comparison of anthocyanins and phenolics in organically and conventionally grown blueberries in selected cultivars. Food Chem..

[39] Roselló-Soto E., Barba F.J., Parniakov O., Galanakis C.M., Lebovka N., Grimi N., Vorobiev E. (2015). High Voltage Electrical Discharges, Pulsed Electric Field, and Ultrasound Assisted Extraction of Protein and Phenolic Compounds from Olive Kernel. Food Bioprocess Technol..

[40] Rajha H.N., Abi-Khattar A.M., El Kantar S., Boussetta N., Lebovka N., Maroun R.G., Louka N., Vorobiev E. (2019). Comparison of aqueous extraction efficiency and biological activities of polyphenols from pomegranate peels assisted by infrared, ultrasound, pulsed electric fields and high-voltage electrical discharges. Innov. Food Sci. Emerg. Technol..

[41] Taruscio T.G., Barney D.L., Exon J. (2004). Content and Profile of Flavanoid and Phenolic Acid Compounds in Conjunction with the Antioxidant Capacity for a Variety of Northwest Vaccinium Berries. J. Agric. Food Chem..

[42] Huang W.Y., Zhang H.C., Liu W.X., Li C.Y. (2012). Survey of antioxidant capacity and phenolic composition of blueberry, blackberry, and strawberry in Nanjing. J. Zhejiang Univ. Sci. B.

[43] Redondo D., Venturini M.E., Luengo E., Raso J., Arias E. (2018). Pulsed electric fields as a green technology for the extraction of bioactive compounds from thinned peach by-products. Innov. Food Sci. Emerg. Technol..

[44] Martín-García B., Tylewicz U., Verardo V., Pasini F., Gómez-Caravaca A.M., Caboni M.F., Dalla Rosa M. (2020). Pulsed electric field (PEF) as pre-treatment to improve the phenolic compounds recovery from brewers’ spent grains. Innov. Food Sci. Emerg. Technol..

[45] Wang Y., Fong S.K., Singh A.P., Vorsa N., Johnson-Cicalese J. (2019). Variation of anthocyanins, proanthocyanidins, flavonols, and organic acids in cultivated and wild diploid blueberry species. HortScience.

[46] Boussetta N., Vorobiev E., Deloison V., Pochez F., Falcimaigne-Cordin A., Lanoisellé J.L. (2011). Valorisation of grape pomace by the extraction of phenolic antioxidants: Application of high voltage electrical discharges. Food Chem..

[47] Trouillas P., Sancho-García J.C., De Freitas V., Gierschner J., Otyepka M., Dangles O. (2016). Stabilizing and modulating color by copigmentation: Insights from theory and experiment. Chem. Rev..

[48] Zheng Y., Wang C.Y., Wang S.Y., Zheng W. (2003). Effect of High-Oxygen Atmospheres on Blueberry Phenolics, Anthocyanins, and Antioxidant Capacity. J. Agric. Food Chem..

[49] Bansal V., Sharma A., Ghanshyam C., Singla M.L. (2014). Optimization and characterization of pulsed electric field parameters for extraction of quercetin and ellagic acid in emblica officinalis juice. J. Food Meas. Charact..

[50] Gharibi Tehrani M., Elhamirad A.H., Azarpazhooh E., Pedramnia A., Sharayei P. (2019). Natural valuable compound extraction from onion by-products using a pulsed electric field. Int. J. Biol. Chem..

[51] Razmara R.S., Daneshfar A., Sahraei R. (2010). Solubility of quercetin in water + methanol and water + ethanol from (292.8 to 333.8). KJ Chem. Eng. Data.

[52] Rababah T.M., Banat F., Rababah A., Ereifej K., Yang W. (2010). Optimization of extraction conditions of total phenolics, antioxidant activities, and anthocyanin of oregano, thyme, terebinth, and pomegranate. J. Food Sci..

[53] Pellegrini N., Colombi B., Salvatore S., Brenna O.V., Galaverna G., Del Rio D., Bianchi M., Bennett R.N., Brighenti F. (2007). Evaluation of antioxidant capacity of some fruit and vegetable foods: Efficiency of extraction of a sequence of solvents. J. Sci. Food Agric..

[54] Boussetta N., Grimi N., Vorobiev E. (2015). Pulsed Electrical Technologies Assisted Polyphenols Extraction from Agricultural Plants and Bioresources: A Review. Int. J. Food Process. Technol..

[55] Cholet C., Delsart C., Petrel M., Gontier E., Grimi N., L’Hyvernay A., Ghidossi R., Vorobiev E., Mietton-Peuchot M., Gény L. (2014). Structural and biochemical changes induced by pulsed electric field treatments on cabernet sauvignon grape berry skins: Impact on cell wall total tannins and polysaccharides. J. Agric. Food Chem..

